# Ensemble learning method for the prediction of new bioactive molecules

**DOI:** 10.1371/journal.pone.0189538

**Published:** 2018-01-12

**Authors:** Lateefat Temitope Afolabi, Faisal Saeed, Haslinda Hashim, Olutomilayo Olayemi Petinrin

**Affiliations:** 1 Department of Physical Sciences, College of Natural Sciences, Al-Hikmah University, Ilorin, Nigeria; 2 College of Computer Science and Engineering, Taibah University, Medina, Saudi Arabia; 3 Information Systems Department, Faculty of Computing, Universiti Teknologi Malaysia, Skudai, Johor, Malaysia; 4 Kolej Yayasan Pelajaran Johor, KM16, Jalan Kulai-Kota Tinggi, Kota Tinggi, Johor, Malaysia; Harbin Institute of Technology Shenzhen Graduate School, CHINA

## Abstract

Pharmacologically active molecules can provide remedies for a range of different illnesses and infections. Therefore, the search for such bioactive molecules has been an enduring mission. As such, there is a need to employ a more suitable, reliable, and robust classification method for enhancing the prediction of the existence of new bioactive molecules. In this paper, we adopt a recently developed combination of different boosting methods (Adaboost) for the prediction of new bioactive molecules. We conducted the research experiments utilizing the widely used MDL Drug Data Report (MDDR) database. The proposed boosting method generated better results than other machine learning methods. This finding suggests that the method is suitable for inclusion among the in silico tools for use in cheminformatics, computational chemistry and molecular biology.

## Background

Virtual screening, which has its roots in cheminformatics, computational chemistry and structural biology [1], is the computation of the similarity between the target (reference structure) and each molecule in a database [2]. It is an established method for the discovery of new biologically active molecules [3]. It is a process whereby, through molecular modeling, each chemical agent in a database is docked into the binding region of each macro molecule target [4]. Docking is the process whereby the best fit for each agent in the binding region of the macromolecular target is calculated [4]. Schneider and Bohm [5] provided a survey of fast automated docking methods, and a detailed study on the calculation of an optimal box size for molecular docking against predicted binding pockets was carried out by Feinstein and Brylinski [6]. Wang et al. [7] extensively reviewed grapheme-based glucose sensors spanning from the period of 2008 to 2015. Huang et al. [8] worked on Drosophila, where Piwi-piRNA was the guiding epigenetic mechanism to target sites. Their work provided insight into the process involved in the recruitment of epigenetic factors to their target sites. Meanwhile, Marinov et al. [9] investigated the work of Huang et al. and discovered that their genome-wide result was not supported by their dataset. The work of Lin et al. [10] confirmed Marinov et al. who stated that the genomic site was not discovered and reaffirmed that the genome RNA polymerase II distribution is influenced by Piwi. Watanabe and Lin reviewed piRNA with respect to some biological processes, and their detailed work can be found in [11]. The science of processing bioactive molecules in important fields, such as lead discovery and compound optimization, has evolved in recent years [12]. The literature has extensively discussed different virtual screening techniques [13–16] and activity prediction approaches [17].

For example, Burden and Winkler [18] introduced the Quantitative Structure-Activity Relationship (QSAR) method as a solution to large datasets and then proposed back propagation (BP) after comparing this method with Multiple Linear Regression (MLR), Principal Component Regression (PCR) and Partial Least Squares (PLS) methods. They applied QSAR to massive data sets derived from combinatorial chemistry and High Throughput Screening (HTS). QSAR involves the prediction of the biological activity of a compound from a vectoral representation of molecular structure [19]. QSAR has been successfully utilized with regards to many drugs and agro-chemical design problems. In Burden and Winkler’s study [18], more information concerning the challenges of QSAR was outlined, and Rogers and Hopfinger [20] solved the problem of building QSAR and Quantity Structure-Property Relationship (QSPR) models using Genetic Function Approximation (GFA). In their work, they disclosed that the secret of the GFA lies in the creation and use of multiple models, rather than the utilization of a single method. Additionally, the unclear QSAR between plant-derived flavones and their inhibiting effects on aurora B kinase (aurB) was established [21].

In the relevant literature, several similarity search methods have been proposed [22]. Sheridan and Kearsley [22] justified the need for many chemical similarity search methods in the early discovery of leads in a drug discovery project. Detailed reviews of chemical similarity searching and virtual screening can be found in Shneider and Bohm [5] and Willett, Barnard and Downs [23].

In this modern era of computational technological advancement, the adoption of machine learning algorithms for the prediction of molecules has been explored. Willet et al. [24] applied the Binary Kernel Discrimination (BKD) approach for the determination of ion channel activity. BKD was introduced and compared with merged similarity search by Harper [25]. Liu et al. [26] developed a model based on the Support Vector Machine, which can be used to automatically produce predictors. This model has a four-in-one function of extracting features, selecting parameters, training models, and cross-validation. This model improves the prediction rate.

A recent survey on the success (to date) and possible opportunities with regards to ligand-based virtual screening in machine learning was performed by Lavecchia [27]. The successes include the development of a large-scale machine learning data protocol, in the work of George et al. [28]; machine learning algorithms in multidimensional analysis of classification performance of compounds, Kurczab and Bojarski [29]; the Naive Bayesian classifier, Kurczab, Smusz and Bojarski [15], Bender et al. [30], and Glick et al. [31]; the Bayesian belief network, Abdo et al. [17], Nidhi et al. [32], and Xia et al. [33]; Support vector machines, Bruce et al. [19] and Buchwald, Ritter and Kramer [34]; Binary kernel discrimination, Willett et al. [24] and Reynolds and Sternberg [25]; the C5 (decision tree), Cao et al. [35]; and Investigational Novel Drug Discovery by Example (INDDEX^TM^), Reynolds and Sterberg [16].

Krasowski and Ekins [36] addressed the challenges faced in correctly detecting and identifying a molecule intake into a class. They utilized cheminformatics to determine the cross reactivity of designer drugs to their available immunoassay (procedure for detecting or measuring specific proteins or other substances through their properties as antigens) [36].

Stumpfe and Bajorath’s study [37] focuses on the practical applications, calculation, and appropriate domain of ligand-based virtual screening. Sherhod et al. [38] generated structural fragmented descriptors by applying a contrast pattern tree mining algorithm. The pattern forms hierarchical clusters of compounds that represent different classes of chemicals. This method was able to identify common toxic features and their classes. Takigawa and Mamitsuka [39] further elaborated on this idea and the procedures for mining frequent sub-graphs for compounds with molecular graphs and chemical compounds.

Smusz et al. [40] adapted virtual screening for their work on the discovery of two structurally new 5-HT_6_R ligands, and Métivier et al. [41] worked on the discovery of structural alerts. In recent research, clustering algorithms have also been used in cheminformatics to discover drugs. A detailed study [42] compares popular clustering techniques, namely, k-means, bisecting k-means and ward clustering. The applications of clustering include QSAR analysis, High Throughput Screening (HTS), and Absorption, Distribution, Metabolism, Elimination and Toxicity (ADMET) prediction [42]. Meanwhile, Pires et al. [43] proposed a novel technique, called pkCSM, to develop predictive models for toxicity properties and small-molecule pharmacokinetics using graph-based signatures [43].

Ensembles have proven to be suitable in improving the performance of a prediction model since they utilize the ability of more than one classifier. They have been used to identify DNA-binding proteins [44] and Piwi-Interacting RNAs [45].

The purpose of our research is to enhance the prediction of bioactive molecules using the boosting algorithm ensemble AdaboostM1 in conjunction with Bagging, Jrip, PART, Random Forest, REPTree and J48 as nominal classifiers. We also compared the performances of the boosting algorithm with a support vector machine classifier called LibSVM (LSVM) [17, 46].

## Materials and methods

### Data sets

Bioactive molecules from both natural products and synthetic compounds are precious sources that provide us with the necessary tools to create new drugs to cure diseases [17]. Molecular fingerprints are representations of chemical structures initially designed to support chemical database substructure searching. Subsequently, their use had been for analysis tasks, such as similarity searching, clustering, and classification. extended connectivity fingerprints (ECFPs) is a recently developed fingerprint methodology specifically designed to identify molecular features significant to molecular activity [47].

Three datasets from ECFP_4 standard molecular descriptors, which were used in previous studies, were used for this study. These datasets were retrieved from the MDDR database. The datasets consist of 8294, 5083, and 8568 instances for DS1, DS2, and DS3, respectively, as shown in Tables 1–3. The quality of prediction was based on these datasets and the validation of the classification of molecules was based on the structure-activity relationship.

**Table 1:** Activity class for dataset DS1.

Activity Index	Activity Class	Activity Molecules	Pairwise Similarity (Mean)
31420	Renin inhibitors	1130	0.573
71523	HIV protease inhibitors	750	0.446
37110	Thrombin inhibitors	803	0.419
31432	Angiotensin II AT1 antagonists	943	0.403
42731	Substance P antagonists	1246	0.339
06233	5HT3 antagonists	752	0.351
06245	5HT reuptake inhibitors	359	0.345
07701	D2 antagonists	395	0.345
06235	5HT1A agonists	827	0.343
78374	Protein kinase C inhibitors	453	0.323
78331	Cyclooxygenase inhibitors	636	0.268

**Table 2:** Activity class for dataset DS2.

Activity Index	Activity Class	Activity Molecules	Pairwise Similarity (Mean)
07707	Adenosine (A1) agonists	207	0.424
07708	Adenosine (A2) agonists	156	0.484
31420	Renin inhibitors	1130	0.584
42710	Monocyclic β-lactams	111	0.596
64100	Cephalosporins	1301	0.512
64200	Carbacephems	158	0.503
64220	Carbapenems	1051	0.414
64300	Penicillin	126	0.444
65000	Antibiotic, macrolide	388	0.673
75755	Vitamin D analogous	455	0.569

**Table 3:** Activity class for dataset DS3.

Activity Index	Activity Class	Activity Molecules	Pairwise Similarity (Mean)
09249	Muscarinic (M1) agonists	900	0.257
12455	NMDA receptor antagonists	1400	0.311
12464	Nitric oxide synthase inhibitors	505	0.237
31281	Dopamine β-hydroxylase inhibitors	106	0.324
43210	Aldose reductase inhibitors	957	0.37
71522	Reverse transcriptase inhibitors	700	0.311
75721	Aromatase inhibitors	636	0.318
78331	Cyclooxygenase inhibitors	636	0.382
78348	Phospholipase A2 inhibitors	617	0.291
78351	Lipoxygenase inhibitors	2111	0.365

The three datasets were pre-processed on the work bench via the following filters: unsupervised, attributes, and Numeric to Nominal. DS 1 contains eleven normal activity classes, DS2 contains ten homogenous (average) activity classes, and DS 2 contains ten heterogeneous activity classes. Tables 1–3 show activity index, activity class, active molecules and pairwise similarity (mean). The active molecules are the number of molecules or peptides belonging to the class and the diversity of classes. The diversity of the class is computed as the mean pairwise Tanimoto similarity score calculated across all pairs of molecules/peptides in the class using ECFP_4.

### Ensemble learning technique

The employment of AdaboostM1 has been discussed in the literature, see for instance [48, 1]. It is a boosting machine learning algorithm [49] that works with another classifier (called the nominal classifier). It works successfully when the nominal classifier in question (also referred to as weak learner) can achieve at least 50% accuracy on its own [49].

AdaboostM1 is an ensemble learning technique and the most well-known of the boosting family of algorithms. The algorithm sequentially trains models, with a new model trained at each round. At the end of each round, misclassified examples are identified and their emphasis is increased in a new training set, which is then fed into the next round and processed to train a new model [50]. The Waikato Environment for Knowledge Analysis (WEKA) software, which is cross-platform software with various machine learning algorithms written in Java, was used to carry out the study. AdaboostM1 is shown in Algorithm 1 (below).

**Algorithm 1**: **AdaboostM1**

**Input**

      Sequence of *m* examples < (*x*_1_,*y_m_*),…,(*x_m_*,*y_m_*) > with labels *y_i_* ∈ *Y* = {1,…,*k*}

       weak learning algorithm **weakLearn**

      integer *T* specifying number of iterations

**Initialize** $D_{1(i)}=\frac{1}{m}$ for all *i*.

**Do for** *t* = 1, 2,…xo, *T*

    1.  Call **weakLearn**, providing it with the distribution *D*_*t*_.

    2.  Get back a hypothesis *h*_*t*_: *X* → *Y*.

    3.  Calculate the error of *h*_*t*_: $\in t=\sum_{i:h_{i}(x_{i})\neq y_{i}}D_{t}(i)$. If $\in t>\frac{1}{2}$, then set *T* = *t*– 1 and abort loop.

    4.  Set *βt* = ∈ *t*/ (1−∈ *t*).

    5.  Update distribution $D_{t}:\;D_{t+1(i)}=\frac{D_{t}(i)}{Z_{t}}\times \left\{ \begin{array}{c} {\beta_{t}}^{ifht(x_{i})=y_{i}}\\ 1_{otherwise} \end{array}\right.$

      where Z_t_ is a normalisation constant (chosen so that D_t+1_ will be a distribution).

**Output**

The final hypothesis: $h_{fin}(x)={arg\;max}_{y\in Y}\;\sum_{t:h_{t}(x)=y}log\frac{1}{\beta_{t}}$

### Experimental design

The need to have a known drug that is classifiable to a specific biological molecular structure is a central part of computational chemistry [51]. In this experiment, we used the extended-connectivity fingerprints (ECFP4) developed by SciTegic [32]. The ECFP4 of MDDR (MDL Drug Data Report) [52] implementation in the test cases is used in this study.

Discovering the optimal parameters for a classifier was a time-consuming task. WEKA-Workbench offers the possibility of automatically finding the best possible setup for the LSVM classifier. The values of 1.0, 0.1, and 0.001 were given to the Cost, Gamma and Epsilon parameters, respectively, while the default values available in WEKA-Workbench were used for the other parameters. In this study, six AdaBoost ensemble classifiers were applied, including AdaBoostM1+Bagging (Ada_Bag), AdaBoostM1+Jrip (Ada_Jrip), AdaBoostM1+J48 (Ada_J48), AdaBoostM1+PART (Ada_PART), AdaBoostM1+RandomForest (Ada_RF), and AdaBoostM1+REPTree (Ada_RT). Subsequently, a ten-fold cross-validation was carried out, and the results were evaluated using sensitivity, specificity, and area under the curve (AUC) measurements.

All experiments were conducted using a personal computer with an Intel® Core ™ i7-4790 CPU 3.60 GHz processor, with 16 GB RAM, and a 64-bit operating system. There are some required settings in the configuration of WEKA to increase the heap size of the memory in the “RunWeka.ini” file under the parameter named “maxheap” with the value of “4096M”. This action supports the processing of the large amount of MDDR datasets being used (the original value was “1024M”).

To validate the performance of each classifier, we used the confusion matrix of the classification results as a measure to compute all the evaluation parameters. The percentage of correctly classified instances from the 10-fold cross validation was used as the measure for the model. In cross validation, the parameter value of 10 was used as the default value. This result suggests that the data set is divided into 10 folds; one fold was used for testing, and the rest were used for training. This process was repeated 10 times so that all folds were used as a test fold once. The error rate is calculated by computing the average of the 10-fold errors.

The area under the receiver operating characteristic curve (AUC), specificity, sensitivity and accuracy were used as the machine learning evaluation methods. These methods are widely used as quality criteria to quantify performance. They are defined as follow:

$$Sensitivity=\frac{TP}{TP+FN}$$

$$Specificity=\frac{TN}{TN+FP}$$

$$Accuracy=\frac{\left(TP+TN\right)}{\left(TP+TN+FP+FN\right)}$$

Where TP = True Positive, FN = False Negative, TN = True Negative, and FP = False Positive.

## Results and discussion

Tables 4–6 display the sensitivity measures (the true positive rates). A number of the AdaBoost ensemble classifiers exhibited the best performance and outperformed the existing best classifier in the discovery of novel drugs where 2 (Ada_Bag and Ada_RF) out of 6 AdaBoost classifiers (Table 4 –DS 1) outperformed the existing best classifier (LSVM).

**Table 4:** Sensitivity measure for the prediction of new bioactive molecules with DS1 (normal sataset).

Class of DS1	Activity Index	LSVM	Ada_Bag	Ada_Jrip	Ada_J48	Ada_PART	Ada_RF	Ada_RT
1	31420	0.978	0.983	0.979	0.980	0.978	**0.985**	0.977
2	71523	0.933	**0.953**	**0.953**	0.945	0.941	0.951	**0.953**
3	37110	0.980	**0.981**	0.978	0.978	0.980	0.976	0.971
4	31432	0.990	0.995	0.990	0.986	0.992	**0.996**	0.989
5	42731	0.986	0.980	0.970	0.970	0.971	**0.990**	0.968
6	6233	0.973	0.979	0.964	0.961	0.951	**0.983**	0.969
7	6245	0.905	**0.916**	0.872	0.855	0.861	0.905	0.886
8	7701	0.851	**0.873**	0.830	0.823	0.810	0.843	0.813
9	6235	0.941	0.949	0.935	0.906	0.900	**0.953**	0.933
10	78374	0.945	0.943	**0.960**	0.932	0.943	0.951	0.916
11	78331	0.970	0.973	0.973	0.947	0.951	**0.980**	0.961
**Mean**		0.950	**0.957**	0.946	0.935	0.934	0.956	0.940

**Table 5:** Sensitivity measure for the prediction of new bioactive molecules with DS2 (homogeneous).

Class of DS2	Activity Index	LSVM	Ada_Bag	Ada_Jrip	Ada_J48	Ada_PART	Ada_RF	Ada_RT
1	07707	0.966	0.961	0.966	0.956	0.956	**0.971**	0.966
2	07708	0.968	0.968	0.962	0.974	0.949	**0.987**	0.949
3	31420	0.995	0.993	0.995	0.992	**0.996**	**0.996**	**0.996**
4	42710	0.982	0.973	0.973	0.982	0.973	0.964	**0.991**
5	64100	0.972	0.978	0.981	0.977	0.975	**0.982**	0.977
6	64200	0.734	0.772	0.715	**0.810**	0.759	0.722	0.759
7	64220	**0.997**	0.996	0.993	0.995	0.995	0.995	0.996
8	64300	0.968	0.952	0.952	**0.976**	0.968	0.968	**0.976**
9	65000	**0.997**	**0.997**	**0.997**	0.995	0.995	0.995	**0.997**
10	75755	**0.996**	**0.996**	0.993	**0.996**	**0.996**	**0.996**	0.993
**Mean**		0.958	0.959	0.953	**0.965**	0.956	0.958	0.960

**Table 6:** Sensitivity measure for the prediction of new bioactive molecules with DS3 (heterogeneous).

Class of DS3	Activity Index	LSVM	Ada_Bag	Ada_Jrip	Ada_J48	Ada_PART	Ada_RF	Ada_RT
1	09249	0.980	0.972	0.979	0.970	0.968	**0.982**	0.974
2	12455	0.955	**0.966**	0.942	0.942	0.946	**0.966**	0.949
3	12464	0.909	0.899	**0.911**	0.907	**0.911**	0.909	0.893
4	31281	**0.972**	0.953	0.934	0.868	0.887	0.915	0.896
5	43210	0.950	**0.956**	0.934	0.947	0.943	**0.956**	0.937
6	71522	0.914	**0.919**	0.916	0.913	0.897	0.909	0.880
7	75721	**0.980**	0.976	0.961	0.945	0.951	0.970	0.956
8	78331	0.838	**0.857**	0.796	0.808	0.832	0.841	0.838
9	78348	0.898	**0.912**	0.878	0.901	0.890	0.891	0.867
10	78351	0.943	0.962	0.958	0.942	0.945	**0.971**	0.949
**Mean**		0.934	**0.937**	0.921	0.914	0.917	0.931	0.914

Table 5 (with DS2) shows that 3 (Ada_Bag, Ada_J48, and Ada_RT) out of 6 AdaBoost classifiers surpassed the LSVM classifier. However, Table 6 (with DS3) illustrates that only 1 (Ada_Bag) out of 6 AdaBoost classifiers surpassed the LSVM classifier.

Tables 7–9 show the specificity measures (the true negative rates), which also demonstrate that a number of AdaBoost classifiers offered the best performance and surpassed the existing best classifier in the discovery of novel drugs, where 2 (Ada_Bag and Ada_RF) out of 6 AdaBoost classifiers (Table 7 –DS1) outperformed the existing best classifier (LSVM).

**Table 7:** Specificity measure for the prediction of new bioactive molecules with DS1 (normal dataset).

Class of DS1	Activity Index	LSVM	Ada_Bag	Ada_Jrip	Ada_J48	Ada_PART	Ada_RF	Ada_RT
1	31420	0.995	**0.997**	**0.997**	0.996	0.978	0.996	**0.997**
2	71523	0.997	0.997	0.997	0.997	0.941	**0.998**	0.996
3	37110	**0.998**	**0.998**	**0.998**	0.996	0.980	**0.998**	0.997
4	31432	**0.999**	**0.999**	**0.999**	0.998	0.992	0.998	**0.999**
5	42731	0.995	**0.996**	0.994	0.992	0.971	0.995	0.993
6	6233	**0.998**	0.997	0.997	0.994	0.951	0.997	0.994
7	6245	0.996	**0.997**	**0.997**	0.996	0.861	**0.997**	0.995
8	7701	0.994	0.996	0.993	0.993	0.810	**0.997**	0.995
9	6235	0.991	**0.993**	0.988	0.990	0.900	0.991	0.990
10	78374	0.998	0.998	0.998	0.997	0.943	**0.999**	0.997
11	78331	**0.997**	0.996	**0.997**	0.994	0.951	**0.997**	0.995
**Mean**		0.996	**0.997**	0.996	0.995	0.934	**0.997**	0.995

**Table 8:** Specificity measure for the prediction of new bioactive molecules with DS2 (homogeneous).

Class of DS2	Activity Index	LSVM	Ada_Bag	Ada_Jrip	Ada_J48	Ada_PART	Ada_RF	Ada_RT
1	07707	**0.999**	**0.999**	**0.999**	**0.999**	**0.999**	**0.999**	**0.999**
2	07708	**0.999**	0.998	**0.999**	**0.999**	0.998	0.998	0.998
3	31420	0.997	**0.998**	**0.998**	0.997	0.997	**0.998**	**0.998**
4	42710	**1.000**	0.999	0.999	0.999	0.999	0.999	0.999
5	64100	0.989	0.990	0.987	**0.992**	0.990	0.989	0.990
6	64200	0.993	0.995	0.995	0.995	0.994	**0.996**	0.995
7	64220	0.998	**0.999**	**0.999**	0.998	**0.999**	0.998	**0.999**
8	64300	0.999	**1.000**	0.999	**1.000**	**1.000**	0.999	**1.000**
9	65000	**1.000**	0.999	0.999	0.999	0.999	**1.000**	**1.000**
10	75755	**1.000**	**1.000**	**1.000**	0.999	**1.000**	**1.000**	**1.000**
**Mean**		0.997	**0.998**	0.997	**0.998**	**0.998**	**0.998**	**0.998**

**Table 9:** Specificity measure for the prediction of new bioactive molecules with DS3 (heterogeneous).

Class of DS3	Activity Index	LSVM	Ada_Bag	Ada_Jrip	Ada_J48	Ada_PART	Ada_RF	Ada_RT
1	09249	**0.997**	0.996	0.996	0.995	0.996	0.996	0.994
2	12455	0.991	0.989	**0.992**	0.987	0.988	0.989	0.985
3	12464	0.996	0.998	0.996	0.995	0.994	**0.999**	0.996
4	31281	0.999	**1.000**	0.999	0.999	0.999	**1.000**	**1.000**
5	43210	0.995	**0.997**	0.996	0.995	0.994	0.996	0.994
6	71522	0.993	0.997	0.998	0.994	0.994	**0.999**	0.995
7	75721	0.997	**0.998**	0.997	0.996	0.996	**0.998**	0.997
8	78331	0.990	0.993	0.991	0.989	0.989	**0.996**	0.989
9	78348	0.992	0.995	0.995	0.994	0.992	**0.996**	0.993
10	78351	**0.976**	0.974	0.956	0.971	0.974	0.965	0.971
**Mean**		0.993	**0.994**	0.992	0.992	0.992	0.993	0.991

Moreover, Table 8 (with DS2) illustrates that 5 (Ada_Bag, Ada_J48, Ada_PART, Ada_RF and Ada_RT) out of 6 AdaBoost classifiers outperformed the LSVM classifier. Table 9 (with DS3) illustrates that only 1 (Ada_Bag) out of 6 AdaBoost classifiers surpassed the LSVM classifier in these specificity measures.

Tables 10–12 display the AUC measures, which also shows that a number of the AdaBoost classifiers offered the best performance and surpassed the existing best classifier in the discovery of novel drugs, where 2 (Ada_Bag and Ada_RF) out of 6 AdaBoost classifiers (Table 10 –DS1) outperformed the existing best classifier (LSVM).

**Table 10:** AUC measure for the prediction of new bioactive molecules with DS1 (normal dataset).

Class of DS1	Activity Index	LSVM	Ada_Bag	Ada_Jrip	Ada_J48	Ada_PART	Ada_RF	Ada_RT
1	31420	0.987	0.990	0.988	0.988	0.987	**0.991**	0.987
2	71523	0.965	**0.975**	**0.975**	0.971	0.970	**0.975**	**0.975**
3	37110	0.989	**0.990**	0.988	0.987	0.988	0.987	0.984
4	31432	0.995	**0.997**	0.995	0.992	0.995	**0.997**	0.994
5	42731	0.991	0.988	0.982	0.981	0.981	**0.993**	0.981
6	6233	0.986	0.988	0.981	0.978	0.973	**0.990**	0.982
7	6245	0.951	**0.957**	0.935	0.926	0.929	0.951	0.941
8	7701	0.923	**0.935**	0.912	0.908	0.902	0.920	0.904
9	6235	0.966	0.971	0.962	0.948	0.945	**0.972**	0.962
10	78374	0.972	0.971	**0.979**	0.965	0.971	0.975	0.957
11	78331	0.984	0.985	0.985	0.971	0.973	**0.989**	0.978
**Mean**		0.973	**0.977**	0.971	0.965	0.965	0.976	0.967

**Table 11:** AUC measure for the prediction of new bioactive molecules with DS2 (homogeneous).

Class of DS2	Activity Index	LSVM	Ada_Bag	Ada_Jrip	Ada_J48	Ada_PART	Ada_RF	Ada_RT
1	07707	0.983	0.980	0.983	0.978	0.978	**0.985**	0.983
2	07708	0.984	0.983	0.981	0.987	0.974	**0.993**	0.974
3	31420	0.996	0.996	**0.997**	0.995	**0.997**	**0.997**	**0.997**
4	42710	0.991	0.986	0.986	0.991	0.986	0.982	**0.995**
5	64100	0.981	0.984	0.984	0.985	0.983	**0.986**	0.984
6	64200	0.864	0.884	0.855	**0.903**	0.877	0.859	0.877
7	64220	**0.998**	**0.998**	0.996	0.997	0.997	0.997	**0.998**
8	64300	0.984	0.976	0.976	**0.988**	0.984	0.984	**0.988**
9	65000	**0.999**	0.998	0.998	0.997	0.997	0.998	**0.999**
10	75755	**0.998**	**0.998**	0.997	**0.998**	**0.998**	**0.998**	0.997
**Mean**		0.977	0.978	0.975	**0.982**	0.977	0.978	0.979

**Table 12:** AUC measure for the prediction of new bioactive molecules with DS3 (heterogeneous).

Class of DS3	Activity Index	LSVM	Ada_Bag	Ada_Jrip	Ada_J48	Ada_PART	Ada_RF	Ada_RT
1	09249	**0.989**	0.984	0.988	0.983	0.982	**0.989**	0.984
2	12455	0.973	**0.978**	0.967	0.965	0.967	**0.978**	0.967
3	12464	0.953	0.949	**0.954**	0.951	0.953	**0.954**	0.945
4	31281	**0.986**	0.977	0.967	0.934	0.943	0.958	0.948
5	43210	0.973	**0.977**	0.965	0.971	0.969	0.976	0.966
6	71522	0.954	**0.958**	0.957	0.954	0.946	0.954	0.938
7	75721	**0.989**	0.987	0.979	0.971	0.974	0.984	0.977
8	78331	0.914	**0.925**	0.894	0.899	0.911	0.919	0.914
9	78348	0.945	**0.954**	0.937	0.948	0.941	0.944	0.930
10	78351	0.960	**0.968**	0.957	0.957	0.960	**0.968**	0.960
**Mean**		0.963	**0.965**	0.956	0.953	0.954	0.962	0.953

Furthermore, Table 11 (with DS2) illustrates that 4 (Ada_Bag, Ada_J48, Ada_RF and Ada_RT) out of 6 AdaBoost classifiers outperformed the LSVM classifier. Table 12 (with DS3) illustrates that there was 1 (Ada_Bag) out of 6 AdaBoost classifiers that surpassed the LSVM classifier for AUC measurements.

From the results illustrated in Tables 4–12, for all three measures (sensitivity, specificity and AUC), it can be seen that in most cases the AdaBoost ensemble classifiers provided better outcomes when compared with LSVM; these ensemble methods built a sequence of base models where each model was constructed based on the performance of the previous model on the training set. In other words, by suitably combining the results of a set of base classifiers, the performance obtained was better than that of any base classifier.

This study used a cut-off value of 0.05 for the significance level (p-value). The p-value was considered significant and capable of providing an overall ranking if p<0.05 and the critical value for chi-square χ^2^ at p = 0.05 for 6 degrees of freedom was 12.59. The degrees of freedom are equal to the total number of algorithms minus 1. In this study, there were 7 algorithms applied (LSVM + six AdaBoost ensemble classifiers), leading to 6 degrees of freedom. The results of Kendall’s W tests are presented in Tables 13–15 (below).

**Table 13:** Rankings of existing best performing classifier (LSVM) and AdaBoost ensemble classifiers, based on Kendall’s W test results using the MDDR dataset by sensitivity measure.

Datasets	W	χ ^2^	p		Ranks						
**DS1**	0.506	33.387	0.000	**Technique**	LSVM	Ada_Bag	Ada_Jrip	Ada_J48	Ada_PART	Ada_RF	Ada_RT
				**Mean Ranks**	4.45	**5.91**	4.18	2.36	2.68	5.86	2.55
**DS2**	0.086	5.176	0.521	**Technique**	LSVM	Ada_Bag	Ada_Jrip	Ada_J48	Ada_PART	Ada_RF	Ada_RT
				**Mean Ranks**	4.4	4.1	3.1	4.1	3.25	4.4	**4.65**
**DS3**	0.397	23.827	0.001	**Technique**	LSVM	Ada_Bag	Ada_Jrip	Ada_J48	Ada_PART	Ada_RF	Ada_RT
				**Mean Ranks**	5.10	**5.70**	3.70	2.55	2.85	5.35	2.75

**Table 14:** Rankings of existing best performing classifier (LSVM) and AdaBoost ensemble classifiers, based on Kendall’s W test results using the MDDR dataset by specificity measure.

Datasets	W	χ^2^	p		Ranks						
**DS1**	0.413	27.287	0.000	**Technique**	LSVM	Ada_Bag	Ada_Jrip	Ada_J48	Ada_PART	Ada_RF	Ada_RT
				**Mean Ranks**	4.64	**5.45**	4.45	2.27	2.73	5.36	3.09
**DS2**	0.043	2.562	0.862	**Technique**	LSVM	Ada_Bag	Ada_Jrip	Ada_J48	Ada_PART	Ada_RF	Ada_RT
				**Mean Ranks**	3.70	4.30	3.90	3.80	3.70	3.95	**4.65**
**DS3**	0.432	25.895	0.000	**Technique**	LSVM	Ada_Bag	Ada_Jrip	Ada_J48	Ada_PART	Ada_RF	Ada_RT
				**Mean Ranks**	4.05	5.65	4.50	2.55	2.55	**5.70**	3.00

**Table 15:** Rankings of existing best performing classifier (LSVM) and AdaBoost ensemble classifiers, based on Kendall’s W test results using the MDDR dataset by AUC measure.

Datasets	W	χ ^2^	p		Ranks						
**DS1**	0.600	39.573	0.000	**Technique**	LSVM	Ada_Bag	Ada_Jrip	Ada_J48	Ada_PART	Ada_RF	Ada_RT
				**Mean Ranks**	4.50	5.91	4.41	2.18	2.27	**6.00**	2.73
**DS2**	0.122	7.293	0.295	**Technique**	LSVM	Ada_Bag	Ada_Jrip	Ada_J48	Ada_PART	Ada_RF	Ada_RT
				**Mean Ranks**	4.35	3.90	2.95	4.30	3.15	4.55	**4.80**
**DS3**	0.486	29.133	0.000	**Technique**	LSVM	Ada_Bag	Ada_Jrip	Ada_J48	Ada_PART	Ada_RF	Ada_RT
				**Mean Ranks**	5.25	**5.85**	3.50	2.55	2.75	5.50	2.60

The analysis in Table 13 shows that Kendall’s coefficients (for DS1 and DS3 using the sensitivity measure) were significant (p<0.05, χ ^2^>12.59) and that the performance of Ada_Bag significantly outperformed all of the other methods. The overall rankings for DS1 were Ada_Bag>Ada_RF> LSVM >Ada_Jrip and Ada_PART>Ada_RT> Ada_J48. For DS3, they were Ada_Bag>Ada_RF> LSVM >Ada_Jrip>Ada_PART>Ada_RT> Ada_J48.

Table 14 illustrates that Kendall’s coefficients (also for DS1 and DS3 using the specificity measure) were significant (p <0.05, χ ^2^> 12.59) and that the performance of Ada_Bag in DS1 and Ada_RF in DS3 significantly outperformed all of the other methods. The overall rankings for DS1 were Ada_Bag>Ada_RF> LSVM >Ada_Jrip>Ada_RT>Ada_PART> Ada_J48. For DS3 the rankings were Ada_RF>Ada_Bag>Ada_Jrip> LSVM >Ada_RT> Ada_J48 >Ada_PART.

Table 15 illustrates that Kendall’s coefficients (also for DS1 and DS3 using the AUC measure) were significant (p <0.05, χ ^2^> 12.59) and that the performance of Ada_RF and Ada_Bag considerably surpassed all of the other methods. The overall rankings for DS1 were Ada_RF>Ada_Bag> LSVM >Ada_Jrip>Ada_RT>Ada_PART> Ada_J48. For DS3 they were Ada_Bag>Ada_RF> LSVM >Ada_Jrip>Ada_PART>Ada_RT> Ada_J48.

In contrast, it can be seen in Tables 13–15 that the results for DS2 using all measures (sensitivity, specificity and AUC) were not significant (p > 0.05, χ ^2^ < 12.59) because the performance of all classifiers in DS2, even though good, were very similar to each other. As such, the differences were not significant.

**Fig 1:**
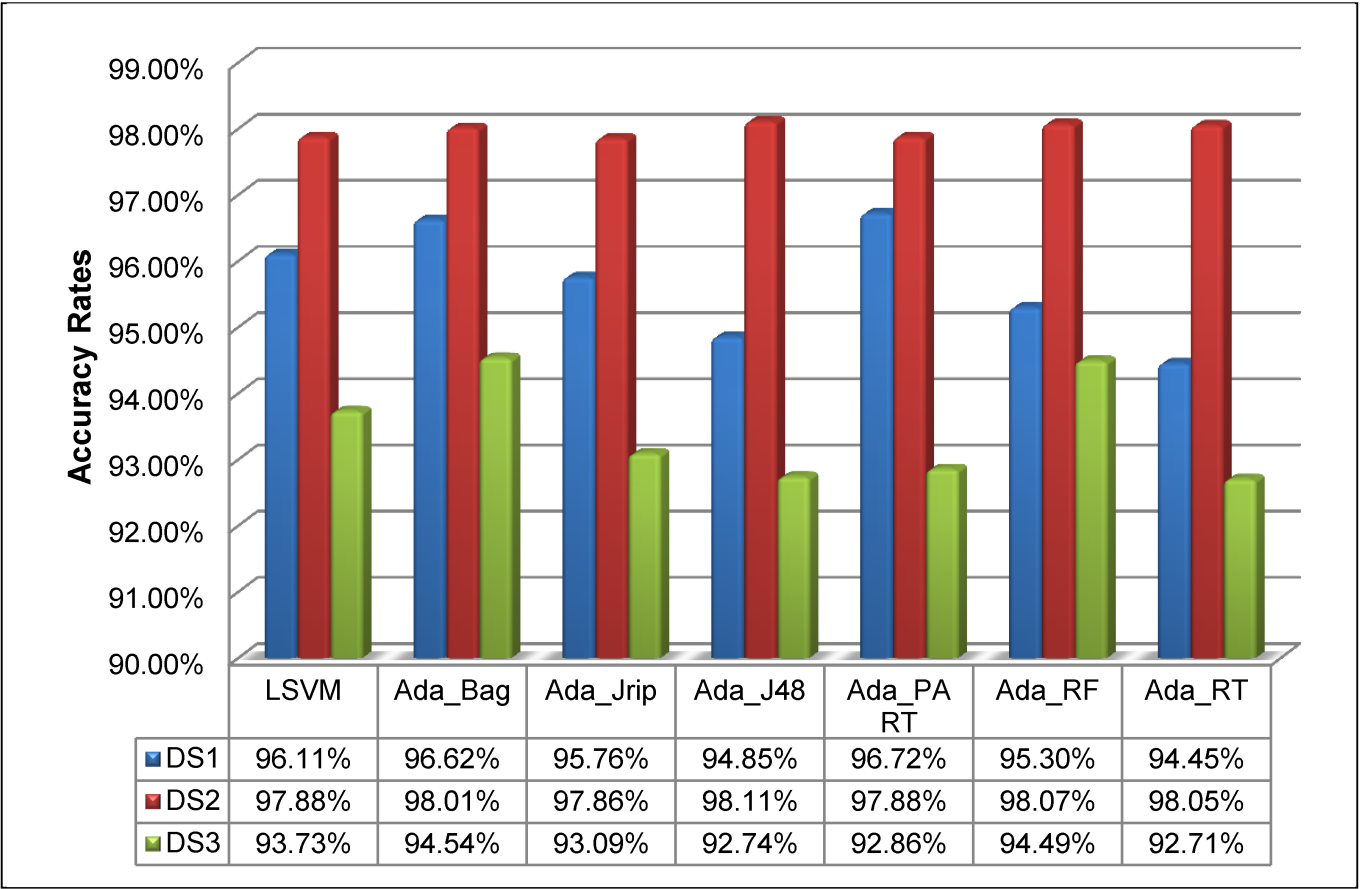
Accuracy rates for the prediction of new bioactive molecules with MDDR (DS1, DS2 and DS3).

Fig 1 (below) illustrates that the highest accuracy was obtained by Ada_PART 96.72% in DS1, Ada_J48 with 98.11% in DS2, and Ada_Bag with 94.54% in DS3. Thus, from the results in Fig 1, we can also conclude that AdaBoost classifiers were able to handle all the datasets.

Most importantly, the results for DS3 (Fig 1) show that using Ada_Bag as the AdaBoost classifier improved the effectiveness of the prediction of new bioactive molecules in highly diverse data when compared to using the existing best classification method (LSVM). The results of DS3 show an accuracy of **94.54%** compared to **93.73%** for LSVM.

In comparison, our proposed methods outperform the method adopted by Liu et al. [44], of which the Liu et al. 2016 method supersedes four other works, as illustrated in their report.

## Conclusions

In this paper, we have presented various machine learning and ensemble methods that were applied to three MDDR benchmark datasets. The results of the experiments illustrate that the incorporation of the boosting algorithm (AdaboostM1), in conjunction with Bagging (Ada_Bag) and Random Forest (Ada_RF) as the nominal classifiers into the in silico discovery of drugs, provides a significant improvement with regard to highly diverse datasets. In future research, other ensemble methods will be examined to see if they improve the effectiveness of the prediction of new bioactive molecules.
